# Antinociceptive Effects of Spinal Manipulative Therapy on Nociceptive Behavior of Adult Rats during the Formalin Test

**DOI:** 10.1155/2015/520454

**Published:** 2015-11-26

**Authors:** Stephen M. Onifer, William R. Reed, Randall S. Sozio, Cynthia R. Long

**Affiliations:** Palmer Center for Chiropractic Research, Palmer College of Chiropractic, 741 Brady Street, Davenport, IA 52803-5214, USA

## Abstract

Optimizing pain relief resulting from spinal manipulative therapies, including low velocity variable amplitude spinal manipulation (LVVA-SM), requires determining their mechanisms. Pain models that incorporate simulated spinal manipulative therapy treatments are needed for these studies. The antinociceptive effects of a single LVVA-SM treatment on rat nociceptive behavior during the commonly used formalin test were investigated. Dilute formalin was injected subcutaneously into a plantar hindpaw. Licking behavior was video-recorded for 5 minutes. Ten minutes of LVVA-SM at 20° flexion was administered with a custom-made device at the lumbar (L5) vertebra of isoflurane-anesthetized experimental rats (*n* = 12) beginning 10 minutes after formalin injection. Hindpaw licking was video-recorded for 60 minutes beginning 5 minutes after LVVA-SM. Control rats (*n* = 12) underwent the same methods except for LVVA-SM. The mean times spent licking the formalin-injected hindpaw of both groups 1–5 minutes after injection were not different. The mean licking time during the first 20 minutes post-LVVA-SM of experimental rats was significantly less than that of control rats (*P* < 0.001). The mean licking times of both groups during the second and third 20 minutes post-LVVA-SM were not different. Administration of LVVA-SM had a short-term, remote antinociceptive effect similar to clinical findings. Therefore, mechanistic investigations using this experimental approach are warranted.

## 1. Introduction

Pain has debilitating effects on function, quality of life, and public health costs [1, 2]. Complementary and integrative health nonpharmacologic mind and body interventions are commonly being used by doctors of chiropractic, osteopathic physicians, and physical therapists to alleviate and manage pain in adults [3]. One of these interventions, spinal manipulative therapy, is broadly divided into spinal manipulation and spinal mobilization approaches [4]. Results from both spinal manipulation and spinal mobilization studies in asymptomatic and symptomatic individuals demonstrate short-term pain relief both remote and local to the anatomical treatment site following a single treatment [5–16].

Recently, the American Pain Society proposed optimizing the use of currently available pain treatments as a goal of their Pain Research Agenda [17]. Additionally, a goal of the National Institutes of Health National Center for Complementary and Integrative Health is to improve complementary and integrative interventions' efficacy for alleviating pain by identifying their mechanisms of action [18]. Essential to achieving these goals is basic science research that uses simulated spinal manipulative therapy techniques with experimental pain models. In the first of such studies, the remote antinociceptive effects of simulated spinal mobilization were determined in adult rats with inflammatory hindpaw nociception [19]. Three minutes of spinal mobilization was delivered to 6 restrained rats at approximately 2 Hertz (Hz) with the experimenter's finger over the lumbar (L5) spinous process beginning 10 minutes after intraplantar hindpaw injection of a mixture of endogenous inflammatory mediators. Pressure pain thresholds of the inflamed hindpaws were increased (decreased mechanical sensitivity) immediately after spinal mobilization ended. However, pressure pain thresholds of the experimental treated group 15 minutes later were not different from those of the control nontreated group. While this peripheral inflammatory pain model allowed for a demonstration of an immediate remote antinociceptive effect following spinal mobilization, investigating the effect's duration requires a model with longer-lasting nociception. Previous remote efficacy and mechanistic studies of knee joint mobilization used ankle joint and gastrocnemius muscle inflammatory pain models where nociception lasted for hours and weeks [20–24]. To build upon the findings from the study of spinal mobilization's antinociceptive effects on inflamed hindpaw nociception [19], we chose to use the formalin test to produce hindpaw inflammatory pain remote to the anatomical treatment site. The formalin test is a commonly used model of peripheral pain that has been performed in rodents, cats, and monkeys [25–27]. This test involves a subcutaneous injection of dilute formalin into a paw that leads to tissue inflammation and nonreflexive, spontaneous nociceptive behaviors [25, 28, 29]. Adult rat nociceptive behaviors of the injected hindpaw are biphasic depending upon whether formalin is subcutaneously injected into the plantar or dorsal paw and the formalin concentration [30–35]. An acute phase of nociceptive behaviors happens within minutes following formalin injection. A quiescent interphase occurs approximately 5 to 10 minutes after injection and is followed by a persistent or tonic phase that lasts for at least 30 minutes.

**Figure 1:**
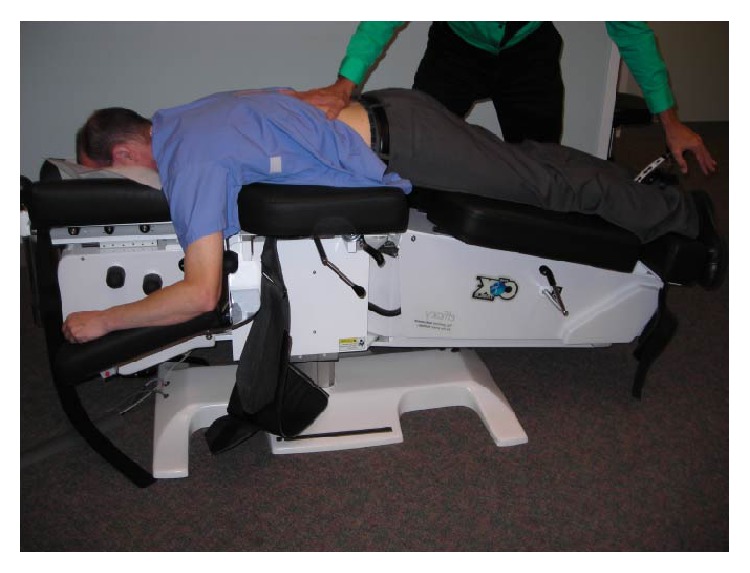
The LVVA-SM technique as performed clinically.

Spinal manipulation can be subdivided into high velocity thrust and low velocity nonthrust approaches based upon the presence or absence of a manipulative thrust, repetitions, and/or the duration of treatment [36]. High velocity low amplitude spinal manipulation (HVLA-SM) is typically delivered with a single, short duration thrust [37]. The frequently utilized [38] low velocity variable amplitude spinal manipulation (LVVA-SM) is applied multiple times without thrust delivery in a controlled cyclical manner to a broader anatomical area [39]. We chose to simulate LVVA-SM since both LVVA-SM and spinal mobilization share clinical similarities and are commonly used across manual therapy disciplines [40]. Moreover, both have been found to be efficacious for managing low back pain [39, 41–44]. Typically during a single treatment, LVVA-SM is delivered to an individual lying prone on a treatment table that is divided into stationary rostral and moveable caudal sections [39, 43, 45–47]. The clinician applies pressure with one hand in a rostral direction to a vertebra's spinous process contact point while controlling the duration, amplitude, and rate of vertebral column flexion via the table's movable section (Figure 1). It is thought that LVVA-SM's applied loads open the intervertebral space and/or foramen leading to changes in the anatomy and physiology of the vertebral column, intervertebral discs, and/or adjacent tissues [39, 43, 48].

The purpose of this study was to determine for the first time the antinociceptive effects of a single simulated LVVA-SM treatment on adult rat remote nociceptive behavior during the commonly used formalin test. Demonstration of antinociceptive effects would warrant using this experimental approach to study mechanisms of spinal manipulative therapy action.

## 2. Materials and Methods

### 2.1. Animals

All methods were approved by the Institutional Animal Care and Use Committee. A total of 32 male Sprague Dawley rats (278–415 g, Harlan Laboratories, Indianapolis, IN, USA) were pair-housed with environment enrichment in a room on a 12-hour light and dark cycle. Access to food and water was ad libitum. One week of housing acclimatization with daily handling occurred before the experiments started.

### 2.2. Effect of LVVA-SM on Nociceptive Behavior during the Formalin Test

To reduce novel environment stress [49, 50], all rats were habituated at the same time of the morning 2 days prior to and the day of the formalin test for 15 minutes in the behavior assessment laboratory (21-22°C, low level of incandescent illumination, and white conversational noise). This was followed by 30 minutes in the observation chamber (23 cm long, 25 cm wide, and 30 cm high, above a mirror angled to aid viewing the hindpaws). The rats were gently placed into a plastic restraint cone (Stoelting Co., Wood Dale, IL) after the last habituation session. Their right hindpaw was held and 50 *μ*L of freshly made formalin (5% v/v of 37% by weight formaldehyde, Fisher Scientific, Fair Lawn, NJ, diluted in sterile saline) was injected from a 1 mL TB syringe through a 30G(1/2)′′ needle into the plantar subcutaneous space at the tori center [25, 33, 51]. The rats were placed into the observation chamber immediately following injection. Behavior was video-recorded with a Sanyo Dual Camera Xacti FH1 for 5 minutes after injection during the acute phase of the formalin test.

**Figure 2:**
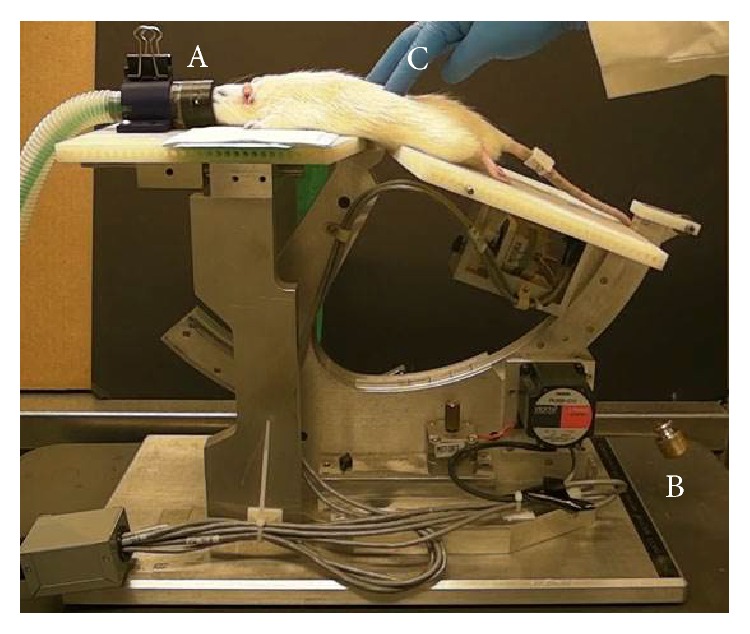
An anesthetized rat undergoing LVVA-SM on a custom-made, motorized device. Mild vertebral column traction was provided using an incisor bar in the anesthesia nose cone (A) and a 100 g weight (B). Mild pressure was applied at the L5 spinous process (C).

During 5–10 minutes after injection (interphase), each rat was placed into an induction chamber and anesthetized with isoflurane (3%, Butler Schein Animal Health, Dublin, OH) in oxygen (2 L/min) for 2 minutes to abolish righting and corneal reflexes. The rat was placed prone on a custom-made device [52] (Figure 2). The device's caudal moveable section was in a neutral position. A water circulating heating pad lays underneath the rat over the device's rostral stationary section. Anesthesia with isoflurane (1.5%) and oxygen (1 L/min) was continued through an anesthesia nose cone attached to the stationary section. A tooth bar within the nose cone was positioned caudal to the incisors to stabilize the rat's head. The rat's L5 vertebra was positioned over the table's fulcrum point. The forelimbs, hindlimbs, and tail were naturally extended away from the body. A silk suture was gently tied over a piece of Velcro brand tape placed around the base of the tail of rats undergoing LVVA-SM (formalin + LVVA-SM, *n* = 12). The suture was draped over a pulley and a 100 g weight was hung from the other end to provide mild traction to the vertebral column. Beginning 10 minutes after formalin injection, LVVA-SM was administered for a clinically relevant duration of 10 minutes [53] at a rate of 0.14 Hz with the device's caudal moveable section cyclically lowered and raised 20° a total of 82 times by a computer-controlled motor. As performed clinically, constant mild pressure was applied in the rostral direction during LVVA-SM by an experimenter's finger placed on the rat's back at the level of the L5 spinous process. Isoflurane in oxygen (1 L/min) was delivered at concentrations of 1.5%, 1%, and 0% during the first 8 minutes, the ninth minute, and the tenth minute of LVVA-SM, respectively. Following 10 minutes of LVVA-SM at 20 minutes after injection, the rats were returned to their cages placed on heating pads to recover from anesthesia. Control rats (formalin + no LVVA-SM, *n* = 12) underwent the exact same methods except that there was no vertebral column traction, cyclic caudal section movement, or manual contact applied by the experimenter. All rats were ambulatory by 25 minutes after injection. At this time, they were placed in the observation chamber and their behavior was video-recorded for 60 minutes. This recording duration was chosen to distinguish between changes in the magnitude or temporal profile of the formalin test's persistent phase [54] after LVVA-SM. In addition, this assessment duration was previously used in efficacy and mechanistic studies of knee joint mobilization on rat hindlimb nociception after joint and muscle inflammation [20, 23, 24]. Each rat was subsequently euthanized by an intraperitoneal injection of Fatal-Plus (0.88 mL/kg, Vortech Pharmaceuticals Ltd., Dearborn, MI) followed by a thoracotomy [55].

Using VLC media player (Version 2.1.2, VideoLAN Organization) and a personal computer, the time (seconds) each rat spent licking the injected hindpaw was quantified during 1–5 minutes after injection (acute phase) and during three 20 minute bins beginning 25 minutes after injection (persistent phase), that is, 5 minutes after being removed from the LVVA-SM device to 65 minutes post-LVVA-SM or no LVVA-SM. We quantified hindpaw licking time due to this nociceptive behavior occurring during both of these phases of the formalin test [31]. Moreover, decreases in hindpaw licking times have been demonstrated to occur in adult rats undergoing other nonpharmacologic interventions administered prior to formalin injection, such as acupuncture, electroacupuncture, and repetitious swimming exercise in warm water [51, 56, 57].

### 2.3. Statistical Methods

Data analyses were done in SAS/STAT (release 9.3; SAS Institute, Inc., Cary, NC). A linear mixed model of the natural log transformation of hindpaw licking time, with a compound symmetry covariance matrix, was used to establish the formalin test. A linear mixed model with an unstructured covariance and terms for group, bin, and group × bin interaction was used to compare mean licking time between the experimental group that received LVVA-SM (formalin + LVVA-SM) and the control group that received no LVVA-SM (formalin + no LVVA-SM) during the formalin test. The model was also used to compare means across time, for adjacent bins, within each group. A *P* < 0.05 was considered statistically significant.

## 3. Results

### 3.1. Formalin Test

To establish the formalin test in our laboratory, rats received a unilateral intraplantar hindpaw injection of either formalin (*n* = 4) or saline (*n* = 4). The rats spent very little time licking the saline-injected hindpaw during 60 minutes after injection. Significantly greater mean time was spent licking the formalin-injected hindpaw than the saline-injected hindpaw during the 1–5 minutes postinjection acute phase of the formalin test (*P* = 0.007). After a quiescent interphase, 5–10 minutes post-formalin injection, the mean licking times of the formalin-injected hindpaw during the test's persistent phase increased and plateaued within 10–25 minutes after injection and then decreased. Significantly greater mean time was spent licking the formalin-injected hindpaw than the saline-injected hindpaw during each 5 minutes for 5–35 minutes after injection (*P* < 0.001). There was a borderline significant difference between the groups at 40 minutes after injection (*P* = 0.07). Mean licking times of the formalin-injected and saline-injected hindpaws were not significantly different after 40 minutes after injection. These results are consistent with those previously reported from a study using the same formalin concentration, injection volume, and injection site [33].

### 3.2. Effect of LVVA-SM on Nociceptive Behavior during the Formalin Test

**Figure 3:**
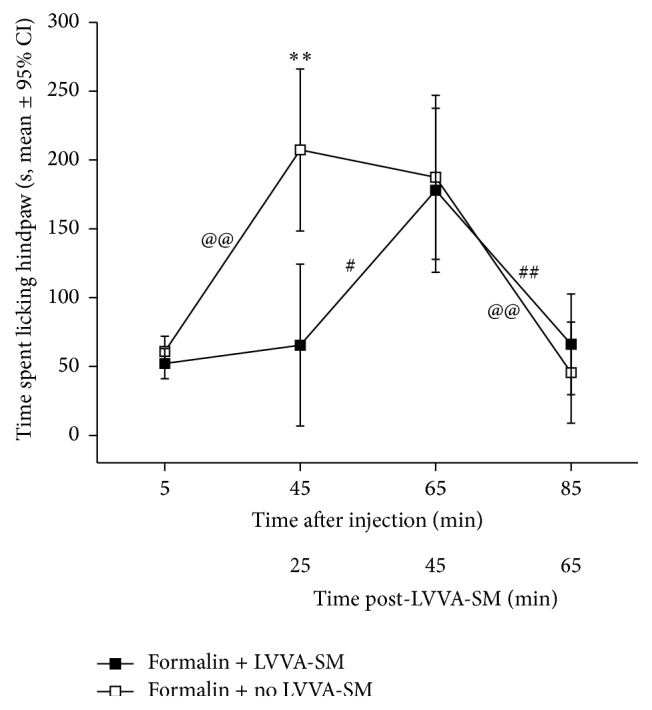
Temporal changes of hindpaw licking behavior following intraplantar formalin injection and either LVVA-SM (formalin + LVVA-SM, *n* = 12) or no LVVA-SM (formalin + no LVVA-SM, *n* = 12). Data are presented as means and 95% confidence intervals (CI) from the linear mixed model. The mean time spent licking the injected hindpaw during the first 20 minutes (min) of 5–65 min post-LVVA-SM by the formalin + LVVA-SM group was less than that of the formalin + no LVVA-SM group (^*∗∗*^ *P* < 0.001 at 25 min post-LVVA-SM). ^@@^ *P* < 0.001, ^#^ *P* = 0.03, and ^##^ *P* < 0.001.

There was a significant group × bin interaction (*F*_3,22_ = 18.7, *P* = 0.003), so the main effects of group and bin time could not be analyzed separately. The mean times spent licking the formalin-injected hindpaw 1–5 minutes after injection were not significantly different between the groups (*P* = 0.25) (Figure 3). The mean time spent licking the injected hindpaw during the first 20 minutes (5–25 minutes post-LVVA-SM) of the formalin + LVVA-SM group was significantly less than that of the formalin + no LVVA-SM group (^*∗∗*^ *P* < 0.001) (Figure 3). The mean licking times of the two groups were not significantly different during the second (*P* = 0.82) and third (*P* = 0.42) 20 minutes post-LVVA-SM.

For the formalin + LVVA-SM group, the mean time spent licking the injected hindpaw was not different between 1–5 minutes after injection and the first 20 minutes post-LVVA-SM (*P* = 0.62), significantly increased during the second 20 minutes (^#^ *P* = 0.03), and then significantly decreased during the third 20 minutes (^##^ *P* < 0.001) (Figure 3). For the formalin + no LVVA-SM group, the mean time spent licking the injected hindpaw significantly increased between 1–5 minutes after injection and the first 20 minutes (^@@^ *P* < 0.001), did not change during the second 20 minutes (*P* = 0.68), and then significantly decreased during the third 20 minutes (^@@^ *P* < 0.001) (Figure 3).

## 4. Discussion

Studies that identify mechanisms of action are needed to optimize pain relief resulting from spinal manipulative therapies. Basic science research that uses experimental pain models in which simulated spinal manual therapy techniques have antinociceptive effects is essential to these investigations. The present study was designed to determine the antinociceptive effects of a single LVVA-SM treatment on adult rat remote nociceptive behavior. Using the established formalin test, the results show that administering LVVA-SM at 20° flexion for 10 minutes at the anesthetized rat's L5 vertebra beginning 10 minutes post-formalin injection significantly decreased hindpaw licking times for the first 20 minutes beginning 5 minutes following the end of treatment.

The results build upon findings from previous basic science and clinical research studies of spinal manipulative therapies that used experimental models of chemically induced inflammation. Three minutes of spinal mobilization was administered at 2 Hz over the nonanesthetized, restrained rat L5 spinous process beginning 10 minutes after intraplantar injection of a mixture of bradykinin, substance P, serotonin, histamine, and prostaglandin E2 into a hindpaw [19]. Pressure pain thresholds of the inflamed hindpaws were increased only when assessed immediately after treatment ended. Administration of HVLA-SM to the human thoracic vertebra 15 minutes after topical application of capsaicin to the forearms immediately reduced evoked secondary hyperalgesia and allodynia as well as perceived pain intensity [58]. When compared to no, sham, and/or placebo interventions, hypoalgesia has also been demonstrated in asymptomatic and symptomatic individuals during assessments performed immediately or 5 minutes following single spinal manipulative therapy treatments [16, 59–68]. In these studies, changes of pressure pain threshold, suprathreshold heat response, temporal sensory summation threshold, and perceived pain intensity were seen remotely at the extremities as well as locally following manipulation or mobilization at the cervical, thoracic, or lumbar vertebrae. Temporal studies also found significant local therapeutic effects on electrical pain tolerance and pressure pain thresholds during assessments 30 seconds–10 minutes and 1–15 minutes following HVLA-SM, respectively [59, 69]. Collectively, these results indicate that a single spinal manipulative therapy treatment provides immediate pain relief both remote and local to the anatomical treatment site that persists at short-term follow-up.

What mechanisms may be responsible for LVVA-SM's significant attenuation of the nociceptive hindpaw licking behavior during the formalin test? Our findings of short-term remote therapeutic effects add to the literature indicating central nervous system (CNS) involvement [12, 15, 52, 70–78]. Studies in adult rats demonstrate that spinal cord and supraspinal neurons are activated following formalin injection into the paw and that the extent of activation parallels the nociceptive licking behavior [79–82]. Therefore, a number of CNS regions playing a role in the formalin-induced nociceptive behaviors likely are involved in LVVA-SM's antinociceptive effects.

In particular, central sensitization is induced within spinal cord dorsal horn neurons during the formalin test's persistent phase [83]. Temporal summation, a measure of dorsal horn neuron central sensitization, has been shown to be immediately inhibited both remotely and locally in asymptomatic and symptomatic persons following spinal manipulation [66, 68, 84, 85]. Reductions in the number of c-fos expressing lumbar spinal cord dorsal horn neurons activated during the formalin test have been observed following acupuncture and electroacupuncture [57, 86–89]. Importantly, these changes were associated with attenuated nociceptive behaviors including hindpaw licking. Therefore, a mechanism of spinal manipulative therapies may involve alterations of pain-induced neuroplastic changes occurring in the spinal cord. These alterations may be due to inhibitory effects on dorsal horn neurons activated by formalin-induced nociception from increased innocuous afferent input generated by LVVA-SM's stimulation of cutaneous and subcutaneous sensory receptors, muscle spindle afferents, and/or Golgi tendon organs [72, 90, 91].

In concert with these inhibitory afferent mechanisms or independently, descending pain inhibitory systems [77, 78] also may be involved. Clinical research using functional magnetic resonance imaging in asymptomatic individuals has demonstrated that decreased remote peripheral pain rating immediately following spinal manipulative therapy was significantly related to reduced neuronal activation in brain regions associated with pain [92]. Basic science research using functional magnetic resonance imaging also showed trends of reduced neuronal activation in brain and lumbar spinal cord regions associated with nociception when knee joint mobilization followed remote ankle joint capsaicin injection [21, 22]. Importantly, the hindpaw secondary mechanical hyperalgesia resulting from ankle joint capsaicin injection was reduced following knee joint mobilization [20]. This antinociceptive effect was prevented or attenuated by intrathecal delivery prior to knee joint mobilization of pharmacological agents that blocked lumbar spinal cord neuron monoamine receptors, but not opioid or GABA receptors [23]. Therefore, involvement of descending pain inhibitory mechanisms using serotonin and noradrenaline is indicated for peripheral manipulative therapy [23]. These mechanisms may be applicable to spinal manipulative therapy as well.

## 5. Limitations

The antinociceptive effect of LVVA-SM during the formalin test's persistent phase was short-term. A future step of our basic science research will be to determine whether LVVA-SM's antinociceptive effects can last longer and their magnitude can be improved. The present study's results may depend upon LVVA-SM's dosage parameters. Eighty-two flexion cycles were administered during the clinically relevant duration of 10 minutes [53]. This 0.14 Hz stimulus rate is slower than the 2 Hz spinal mobilization used by Grayson and colleagues [19] for 3 minutes in rats with inflamed hindpaws. Clinical research of spinal mobilization in asymptomatic individuals found that stimulus rates of 1 and 2 Hz delivered to lumbar vertebrae for 3 minutes led to similar immediate increases in pain pressure thresholds assessed both remotely and locally to the treatment site [93]. A stimulus rate of 2 Hz administered to cervical vertebrae for 3 minutes produced a greater increase in skin conductance, but not skin temperature, than 0.5 Hz for 10 minutes after treatment [94]. Furthermore, basic science research of peripheral knee joint mobilization demonstrated that durations of 9 and 15, but not 3, minutes produced antinociception measured at the hindpaw following remote ankle joint nociception [20]. These differing results indicate that systematic evaluation of LVVA-SM's dosage parameters and their antinociceptive effects is needed.

The transient duration of nociceptive behaviors during the formalin test's persistent phase limits its further use to mechanistic studies of LVVA-SM's short-term remote effect. Investigation of LVVA-SM's long-term remote antinociceptive effects will require experimental models where nociceptive behaviors last for hours or days, such as in those of peripheral joint or muscle pain in which long-term antinociceptive effects of knee joint mobilization have been found [20, 23, 24]. A model of chronic peripheral neuropathic pain [95] may be useful as well.

The individual assessing the time spent licking the hindpaw was not blinded to group assignment. Therefore, the risk of assessor bias could be considered a limitation of our study despite hindpaw licking time being an objective outcome measure. It was not possible to perform LVVA-SM in conscious rats due to the stress that may result and stress-induced analgesia [96]. While spinal manipulative therapies can be clinically delivered under anesthesia [97] and anesthesia has been used during manual therapy in previous studies of laboratory rats [20, 86], administering LVVA-SM to anesthetized rats may be a limitation of our study. However, we included an anesthetized control group to account for this possibility. Our choice of isoflurane was based upon the finding that rat nociceptive behavior was modestly attenuated during the formalin test's persistent phase following isoflurane administration [98].

## 6. Conclusion

The present study shows that lumbar LVVA-SM administration to adult rats during the formalin test is capable of reducing remote nociceptive behavior for a short-term. Since this result is similar to clinical research findings, mechanistic investigations of LVVA-SM's short-term remote antinociceptive effect can using the formalin test are warranted.

## References

[1] Hoy D., March L., Brooks P. (2014). The global burden of low back pain: estimates from the Global Burden of Disease 2010 Study. *Annals of the Rheumatic Diseases*.

[2] Hoy D., March L., Woolf A. (2014). The global burden of neck pain: estimates from the global burden of disease 2010 study. *Annals of the Rheumatic Diseases*.

[3] Clarke T. C., Black L. I., Stussman B. J., Barnes P. M., Nahin R. L. (2015). Trends in the use of complementary health approaches among adults: United States, 2002–2012. *National Health Statistics Reports*.

[4] Bolton P. S., Budgell B. S. (2005). Spinal manipulation and spinal mobilization influence different axial sensory beds. *Medical Hypotheses*.

[5] Coronado R. A., Bialosky J. E., Cook C. E. (2010). The temporal effects of a single session of high-velocity, low-amplitude thrust manipulation on subjects with spinal pain. *Physical Therapy Reviews*.

[6] Coronado R. A., Gay C. W., Bialosky J. E., Carnaby G. D., Bishop M. D., George S. Z. (2012). Changes in pain sensitivity following spinal manipulation: a systematic review and meta-analysis. *Journal of Electromyography and Kinesiology*.

[7] Furlan A. D., Yazdi F., Tsertsvadze A. (2012). A systematic review and meta-analysis of efficacy, cost-effectiveness, and safety of selected complementary and alternative medicine for neck and low-back pain. *Evidence-Based Complementary and Alternative Medicine*.

[8] Goertz C. M., Pohlman K. A., Vining R. D., Brantingham J. W., Long C. R. (2012). Patient-centered outcomes of high-velocity, low-amplitude spinal manipulation for low back pain: a systematic review. *Journal of Electromyography and Kinesiology*.

[9] Hegedus E. J., Goode A., Butler R. J., Slaven E. (2011). The neurophysiological effects of a single session of spinal joint mobilization: does the effect last?. *Journal of Manual & Manipulative Therapy*.

[10] Kuczynski J. J., Schwieterman B., Columber K., Knupp D., Shaub L., Cook C. E. (2012). Effectiveness of physical therapist administered spinal manipulation for the treatment of low back pain: a systematic review of the literature. *International Journal of Sports Physical Therapy*.

[11] Millan M., Leboeuf-Yde C., Budgell B., Amorim M.-A. (2012). The effect of spinal manipulative therapy on experimentally induced pain: a systematic literature review. *Chiropractic & Manual Therapies*.

[12] Schmid A., Brunner F., Wright A., Bachmann L. M. (2008). Paradigm shift in manual therapy? Evidence for a central nervous system component in the response to passive cervical joint mobilisation. *Manual Therapy*.

[13] Scholten-Peeters G. G. M., Thoomes E., Konings S. (2013). Is manipulative therapy more effective than sham manipulation in adults?: a systematic review and meta-analysis. *Chiropractic and Manual Therapies*.

[14] Slaven E. J., Goode A. P., Coronado R. A., Poole C., Hegedus E. J. (2013). The relative effectiveness of segment specific level and non-specific level spinal joint mobilization on pain and range of motion: results of a systematic review and meta-analysis. *Journal of Manual and Manipulative Therapy*.

[15] Vernon H. (2000). Qualitative review of studies of manipulation-induced hypoalgesia. *Journal of Manipulative and Physiological Therapeutics*.

[16] Vicenzino B., Collins D., Wright A. (1996). The initial effects of a cervical spine manipulative physiotherapy treatment on the pain and dysfunction of lateral epicondylalgia. *Pain*.

[17] Gereau R. W., Sluka K. A., Maixner W. (2014). A pain research agenda for the 21st century. *Journal of Pain*.

[18] U. S. Department of Health and Human Services. National Institutes of Health NCCAM (2011). *Third Strategic Plan 2011–2015: Exploring the Science of Complementary and Alternative Medicine*.

[19] Grayson J. E., Barton T., Cabot P. J., Souvlis T. (2012). Spinal manual therapy produces rapid onset analgesia in a rodent model. *Manual Therapy*.

[20] Sluka K. A., Wright A. (2001). Knee joint mobilization reduces secondary mechanical hyperalgesia induced by capsaicin injection into the ankle joint. *European Journal of Pain*.

[21] Malisza K. L., Gregorash L., Turner A. (2003). Functional MRI involving painful stimulation of the ankle and the effect of physiotherapy joint mobilization. *Magnetic Resonance Imaging*.

[22] Malisza K. L., Stroman P. W., Turner A., Gregorash L., Foniok T., Wright A. (2003). Functional MRI of the rat lumbar spinal cord involving painful stimulation and the effect of peripheral joint mobilization. *Journal of Magnetic Resonance Imaging*.

[23] Skyba D. A., Radhakrishnan R., Rohlwing J. J., Wright A., Sluka K. A. (2003). Joint manipulation reduces hyperalgesia by activation of monoamine receptors but not opioid or GABA receptors in the spinal cord. *Pain*.

[24] Sluka K. A., Skyba D. A., Radhakrishnan R., Leeper B. J., Wright A. (2006). Joint mobilization reduces hyperalgesia associated with chronic muscle and joint inflammation in rats. *Journal of Pain*.

[25] Dubuisson D., Dennis S. G. (1977). The formalin test: a quantitative study of the analgesic effects of morphine, meperidine, and brain stem stimulation in rats and cats. *Pain*.

[26] Alreja M., Mutalik P., Nayar U., Manchanda S. K. (1984). The formalin test: a tonic pain model in the primate. *Pain*.

[27] Hunskaar S., Fasmer O. B., Hole K. (1985). Formalin test in mice, a useful technique for evaluating mild analgesics. *Journal of Neuroscience Methods*.

[28] Tjølsen A., Berge O.-G., Hunskaar S., Rosland J. H., Hole K. (1992). The formalin test: an evaluation of the method. *Pain*.

[29] Gregory N. S., Harris A. L., Robinson C. R., Dougherty P. M., Fuchs P. N., Sluka K. A. (2013). An overview of animal models of pain: disease models and outcome measures. *The Journal of Pain*.

[30] Coderre T. J., Fundytus M. E., McKenna J. E., Dalal S., Melzack R. (1993). The formalin test: a validation of the weighted-scores method of behavioural pain rating. *Pain*.

[31] Abbott F. V., Franklin K. B. J., Westbrook R. F. (1995). The formalin test: scoring properties of the first and second phases of the pain response in rats. *Pain*.

[32] Aloisi A. M., Albonetti M. E., Carli G. (1995). Behavioural effects of different intensities of formalin pain in rats. *Physiology and Behavior*.

[33] Okuda K., Sakurada C., Takahashi M., Yamada T., Sakurada T. (2001). Characterization of nociceptive responses and spinal releases of nitric oxide metabolites and glutamate evoked by different concentrations of formalin in rats. *Pain*.

[34] Yaksh T. L., Ozaki G., McCumber D. (2001). An automated flinch detecting system for use in the formalin nociceptive bioassay. *Journal of Applied Physiology*.

[35] Lee I. O., Jeong Y. S. (2002). Effects of different concentrations of formalin on paw edema and pain behaviors in rats. *Journal of Korean Medical Science*.

[36] Cleland J. A., Fritz J. M., Kulig K. (2009). Comparison of the effectiveness of three manual physical therapy techniques in a subgroup of patients with low back pain who satisfy a clinical prediction rule: a randomized clinical trial. *Spine*.

[37] Evans D. W., Lucas N. (2010). What is ‘manipulation’? A reappraisal. *Manual Therapy*.

[38] Christensen M. G., Hyland J. K., Goertz C. M., Kollasch M. W. (2015). *Practice Analysis of Chiropractic*.

[39] Hondras M. A., Long C. R., Cao Y., Rowell R. M., Meeker W. C. (2009). A randomized controlled trial comparing 2 types of spinal manipulation and minimal conservative medical care for adults 55 years and older with subacute or chronic low back pain. *Journal of Manipulative and Physiological Therapeutics*.

[40] Wilder D. G., Vining R. D., Pohlman K. A. (2011). Effect of spinal manipulation on sensorimotor functions in back pain patients: study protocol for a randomised controlled trial. *Trials*.

[41] Goodsell M., Lee M., Latimer J. (2000). Short-term effects of lumbar posteroanterior mobilization in individuals with low-back pain. *Journal of Manipulative and Physiological Therapeutics*.

[42] Cambron J. A., Gudavalli M. R., Hedeker D. (2006). One-year follow-up of a randomized clinical trial comparing flexion distraction with an exercise program for chronic low-back pain. *Journal of Alternative and Complementary Medicine*.

[43] Gudavalli M. R., Cambron J. A., McGregor M. (2006). A randomized clinical trial and subgroup analysis to compare flexion-distraction with active exercise for chronic low back pain. *European Spine Journal*.

[44] Shum G. L., Tsung B. Y., Lee R. Y. (2013). The immediate effect of posteroanterior mobilization on reducing back pain and the stiffness of the lumbar spine. *Archives of Physical Medicine and Rehabilitation*.

[45] Gay R. E., Bronfort G., Evans R. L. (2005). Distraction manipulation of the lumbar spine: a review of the literature. *Journal of Manipulative and Physiological Therapeutics*.

[46] Cox J. M. (2011). *Low Back Pain: Mechanism, Diagnosis, Treatment*.

[47] Gudavalli M. R., Cox J. M. (2015). Real-time force feedback during flexion-distraction procedure for low back pain: a pilot study. *The Journal of the Canadian Chiropractic Association*.

[48] Gudavalli M. R., Potluri T., Carandang G. (2013). Intradiscal pressure changes during manual cervical distraction: a cadaveric study. *Evidence-Based Complementary and Alternative Medicine*.

[49] Abbott F. V., Franklin K. B. J., Connell B. (1986). The stress of a novel environment reduces formalin pain: possible role of serotonin. *European Journal of Pharmacology*.

[50] Capone F., Aloisi A. M. (2004). Refinement of pain evaluation techniques. The formalin test. *Annali dell'Istituto Superiore di Sanità*.

[51] Kuphal K. E., Fibuch E. E., Taylor B. K. (2007). Extended swimming exercise reduces inflammatory and peripheral neuropathic pain in rodents. *The Journal of Pain*.

[52] Henderson C. N. R. (2012). The basis for spinal manipulation: chiropractic perspective of indications and theory. *Journal of Electromyography and Kinesiology*.

[53] Cambron J. A., Schneider M., Dexheimer J. M. (2014). A pilot randomized controlled trial of flexion-distraction dosage for chiropractic treatment of lumbar spinal stenosis. *Journal of Manipulative and Physiological Therapeutics*.

[54] Taylor B. K., Peterson M. A., Basbaum A. I. (1997). Early nociceptive events influence the temporal profile, but not the magnitude, of the tonic response to subcutaneous formalin: effects with remifentanil. *Journal of Pharmacology and Experimental Therapeutics*.

[55] Onifer S. M., Quintero J. E., Gerhardt G. A. (2012). Cutaneous and electrically evoked glutamate signaling in the adult rat somatosensory system. *Journal of Neuroscience Methods*.

[56] Yi M., Zhang H., Lao L., Xing G.-G., Wan Y. (2011). Anterior cingulate cortex is crucial for contra- but not ipsi-lateral electro-acupuncture in the formalin-induced inflammatory pain model of rats. *Molecular Pain*.

[57] Chang K. H., Bai S. J., Lee Y., Lee B. H. (2014). Effects of acupuncture stimulation at different acupoints on formalin-induced pain in rats. *Korean Journal of Physiology and Pharmacology*.

[58] Mohammadian P., Gonsalves A., Tsai C., Hummel T., Carpenter T. (2004). Areas of capsaicin-induced secondary hyperalgesia and allodynia are reduced by a single chiropractic adjustment: a preliminary study. *Journal of Manipulative and Physiological Therapeutics*.

[59] Terrett A. C. J., Vernon H. (1984). Manipulation and pain tolerance. A controlled study of the effect of spinal manipulation on paraspinal cutaneous pain tolerance levels. *American Journal of Physical Medicine*.

[60] Sterling M., Jull G., Wright A. (2001). Cervical mobilisation: concurrent effects on pain, sympathetic nervous system activity and motor activity. *Manual Therapy*.

[61] Fernández-De-Las-Peñas C., Pérez-de-Heredia M., Brea-Rivero M., Miangolarra-Page J. C. (2007). Immediate effects on pressure pain threshold following a single cervical spine manipulation in healthy subjects. *Journal of Orthopaedic and Sports Physical Therapy*.

[62] Fernández-de-las-Peñas C., Alonso-Blanco C., Cleland J. A., Rodríguez-Blanco C., Alburquerque-Sendín F. (2008). Changes in pressure pain thresholds over C5-C6 zygapophyseal joint after a cervicothoracic junction manipulation in healthy subjects. *Journal of Manipulative and Physiological Therapeutics*.

[63] Fernández-Carnero J., Fernández-de-las-Peñas C., Cleland J. A. (2008). Immediate hypoalgesic and motor effects after a single cervical spine manipulation in subjects with lateral epicondylalgia. *Journal of Manipulative and Physiological Therapeutics*.

[64] McClatchie L., Laprade J., Martin S., Jaglal S. B., Richardson D., Agur A. (2009). Mobilizations of the asymptomatic cervical spine can reduce signs of shoulder dysfunction in adults. *Manual Therapy*.

[65] Bicalho E., Palma Setti J. A., Macagnan J., Rivas Cano J. L., Manffra E. F. (2010). Immediate effects of a high-velocity spine manipulation in paraspinal muscles activity of nonspecific chronic low-back pain subjects. *Manual Therapy*.

[66] Bishop M. D., Beneciuk J. M., George S. Z. (2011). Immediate reduction in temporal sensory summation after thoracic spinal manipulation. *Spine Journal*.

[67] de Camargo V. M., Alburquerque-Sendín F., Bérzin F., Stefanelli V. C., Rodrigues de Souza D. P., Fernández-de-las-Peñas C. (2011). Immediate effects on electromyographic activity and pressure pain thresholds after a cervical manipulation in mechanical neck pain: a randomized controlled trial. *Journal of Manipulative and Physiological Therapeutics*.

[68] Bialosky J. E., George S. Z., Horn M. E., Price D. D., Staud R., Robinson M. E. (2014). Spinal manipulative therapy-specific changes in pain sensitivity in individuals with low back pain (NCT01168999). *Journal of Pain*.

[69] Srbely J. Z., Vernon H., Lee D., Polgar M. (2013). Immediate effects of spinal manipulative therapy on regional antinociceptive effects in myofascial tissues in healthy young adults. *Journal of Manipulative and Physiological Therapeutics*.

[70] Wright A. (1995). Hypoalgesia post-manipulative therapy: a review of a potential neurophysiological mechanism. *Manual Therapy*.

[71] Evans D. W. (2002). Mechanisms and effects of spinal high-velocity, low-amplitude thrust manipulation: previous theories. *Journal of Manipulative and Physiological Therapeutics*.

[72] Pickar J. G. (2002). Neurophysiological effects of spinal manipulation. *Spine Journal*.

[73] Boal R. W., Gillette R. G. (2004). Central neuronal plasticity, low back pain and spinal manipulative therapy. *Journal of Manipulative and Physiological Therapeutics*.

[74] Bialosky J. E., Bishop M. D., Price D. D., Robinson M. E., George S. Z. (2009). The mechanisms of manual therapy in the treatment of musculoskeletal pain: a comprehensive model. *Manual Therapy*.

[75] Clark B. C., Thomas J. S., Walkowski S. A., Howell J. N. (2012). The biology of manual therapies. *Journal of the American Osteopathic Association*.

[76] Haavik H., Murphy B. (2012). The role of spinal manipulation in addressing disordered sensorimotor integration and altered motor control. *Journal of Electromyography and Kinesiology*.

[77] Sparks C., Cleland J., Elliott J., Strubhar A. (2013). Supraspinal structures may be associated with hypoalgesia following thrust manipulation to the spine: a review of the literature. *Physical Therapy Reviews*.

[78] Savva C., Giakas G., Efstathiou M. (2014). The role of the descending inhibitory pain mechanism in musculoskeletal pain following high-velocity, low amplitude thrust manipulation. A review of the literature. *Journal of Back and Musculoskeletal Rehabilitation*.

[79] Pórszász R., Beckmann N., Bruttel K., Urban L., Rudin M. (1997). Signal changes in the spinal cord of the rat after injection of formalin into the hindpaw: characterization using functional magnetic resonance imaging. *Proceedings of the National Academy of Sciences of the United States of America*.

[80] Porro C. A., Cavazzuti M., Lui F., Giuliani D., Pellegrini M., Baraldi P. (2003). Independent time courses of supraspinal nociceptive activity and spinally mediated behavior during tonic pain. *Pain*.

[81] Fukuda T., Watanabe K., Hisano S., Toyooka H. (2006). Licking and c-fos expression in the dorsal horn of the spinal cord after the formalin test. *Anesthesia and Analgesia*.

[82] Asante C. O., Wallace V. C., Dickenson A. H. (2009). Formalin-induced behavioural hypersensitivity and neuronal hyperexcitability are mediated by rapid protein synthesis at the spinal level. *Molecular Pain*.

[83] Coderre T. J., Melzack R. (1992). The contribution of excitatory amino acids to central sensitization and persistent nociception after formalin-induced tissue injury. *Journal of Neuroscience*.

[84] George S. Z., Bishop M. D., Bialosky J. E., Zeppieri G., Robinson M. E. (2006). Immediate effects of spinal manipulation on thermal pain sensitivity: an experimental study. *BMC Musculoskeletal Disorders*.

[85] Bialosky J. E., Bishop M. D., Robinson M. E., Zeppieri G., George S. Z. (2009). Spinal manipulative therapy has an immediate effect on thermal pain sensitivity in people with low back pain: a randomized controlled trial. *Physical Therapy*.

[86] Wen Y.-R., Yeh G.-C., Shyu B.-C. (2007). A minimal stress model for the assessment of electroacupuncture analgesia in rats under halothane. *European Journal of Pain*.

[87] Kim H., Shim I., Yi S. H., Lee H., Lim H.-S., Hahm D.-H. (2009). Warm needle acupuncture at Pungsi (GB31) has an enhanced analgesic effect on formalin-induced pain in rats. *Brain Research Bulletin*.

[88] Kim G. H., Yeom M., Yin C. S. (2010). Acupuncture manipulation enhances anti-nociceptive effect on formalin-induced pain in rats. *Neurological Research*.

[89] Chang K.-H., Won R., Shim I., Lee H., Lee B. H. (2012). Effects of electroacupuncture at BL60 on formalin-induced pain in rats. *Evidence-Based Complementary and Alternative Medicine*.

[90] Melzack R., Wall P. D. (1965). Pain mechanisms: a new theory. *Science*.

[91] Bulbulian R., Burke J., Dishman J. D. (2002). Spinal reflex excitability changes after lumbar spine passive flexion mobilization. *Journal of Manipulative and Physiological Therapeutics*.

[92] Sparks C., Cleland J. A., Elliott J. M., Zagardo M., Liu W.-C. (2013). Using functional magnetic resonance imaging to determine if cerebral hemodynamic responses to pain change following thoracic spine thrust manipulation in healthy individuals. *Journal of Orthopaedic and Sports Physical Therapy*.

[93] Willett E., Hebron C., Krouwel O. (2010). The initial effects of different rates of lumbar mobilisations on pressure pain thresholds in asymptomatic subjects. *Manual Therapy*.

[94] Chiu T. W., Wright A. (1996). To compare the effects of different rates of application of a cervical mobilisation technique on sympathetic outflow to the upper limb in normal subjects. *Manual Therapy*.

[95] Decosterd I., Woolf C. J. (2000). Spared nerve injury: an animal model of persistent peripheral neuropathic pain. *Pain*.

[96] Butler R. K., Finn D. P. (2009). Stress-induced analgesia. *Progress in Neurobiology*.

[97] Digiorgi D. (2013). Spinal manipulation under anesthesia: a narrative review of the literature and commentary. *Chiropractic and Manual Therapies*.

[98] Abram S. E., Yaksh T. L. (1993). Morphine, but not inhalation anesthesia, blocks post-injury facilitation. The role of preemptive suppression of afferent transmission. *Anesthesiology*.

